# How Does Urban Green Space Impact Residents’ Mental Health: A Literature Review of Mediators

**DOI:** 10.3390/ijerph182211746

**Published:** 2021-11-09

**Authors:** Kaili Chen, Tianzheng Zhang, Fangyuan Liu, Yingjie Zhang, Yan Song

**Affiliations:** 1 School of Economics and Management, Beijing Forestry University, Beijing 100083, China; chenkaili@bjfu.edu.cn (K.C.); zh_tz@bjfu.edu.cn (T.Z.); lfy2020@bjfu.edu.cn (F.L.); 2 National Academy of Economics and Trade for Forestry and Grassland, Beijing Forestry University, Beijing 100083, China; 3 The Department of City and Regional Planning, University of North Carolina, Chapel Hill, NC 27599, USA; ys@email.unc.edu

**Keywords:** greenery, urban forest, psychological relaxation, intermediary factors, influencing path

## Abstract

In recent years, the interest in the relationship between urban green space and residents’ mental health has gradually risen. A number of researchers have investigated the causal relationship and possible mediators between the two, although few have summarized these mediators. For this reason, we searched for relevant studies and filtered them by criteria and quality score, and analyzed the mediators and paths of the impact of urban green space on residents’ mental health. The mediators can be divided into environmental factors, outdoor activity, and social cohesion. From the perspective of heterogeneity, both individual characteristics (e.g., age and gender) and group characteristics (e.g., level of urban development and urban density) of residents are considered to be the cause of various mediating effects. Types of urban green space tend to affect residents’ mental health through different paths. Furthermore, this review discusses the details of each part under the influence paths. Finally, the policy implications for urban green space planning from three mediator levels are put forward based on an analysis of the situation in different countries.

## 1. Introduction

In recent years, the built environment and human mental health have attracted extensive attention from the international community. The World Health Organization has pointed out that the Healthy Cities movement has become a pioneer in urban development and transformation, providing an impetus to the creation of a healthier and friendlier urban environment as well as maintaining human mental health and well-being [1,2]. As an important part of urban built environment, urban green space has long been recognized in the fields of promoting residents’ mental health.

In general, certain theoretical achievements have been made in the research on the correlation between urban green space and residents’ mental health. A series of studies have confirmed that urban green space is closely related to the mental health of residents. Lee et al. [3] pointed out there is a causal link between various indicators of mental health and urban green space, according to the meta-analysis. Urban green space can improve residents’ mental health by stabilizing emotions and releasing stress [4]. Using the national representative longitudinal samples of British residents, White et al. [5] found that residents living in urban areas with a relatively high greening level have a lower average mental stress and higher life satisfaction. Volker et al. [6] also obtained similar results on this topic in Germany.

Based on the benefits of urban green space on mental health, it is of great significance to clarify the mechanism of urban green space on mental health. The main challenges that still need to be addressed in this research field are the causes and mediators of green space’s beneficial effects [7,8]. However, the mediators of this relationship are not clearly defined, and many of the mediators currently proposed are likely to overlap conceptually with measures of urban green space or mental health (such as green space quality and stress). Therefore, this paper aims to summarize the current mediators and identify the impact paths of different mediators. Furthermore, this article specifically analyzes the heterogeneous effects of the above-mentioned influences, considering not only different types of green spaces, but also residents with different socioeconomic characteristics. These findings should make an important contribution to the field of causality analysis between urban green space and residents’ mental health, as well as demand-oriented urban green space planning and management.

The structure of the remainder of this article is as follows: First, we summarize the possible mediating factors and their influencing paths between urban green space and residents’ mental health by searching for relevant studies around the world. Literature search and selection are carried out in Section 2. Next, a comprehensive analysis and discussion of the limitations associated with each part under the influence paths is provided in Section 3 and Section 4. Finally, based on an analysis of the situation in other countries, we put forward some policy implications at different mediator levels from the perspective of urban administrators in Section 5.

## 2. Materials and Methods

**Figure 1:**
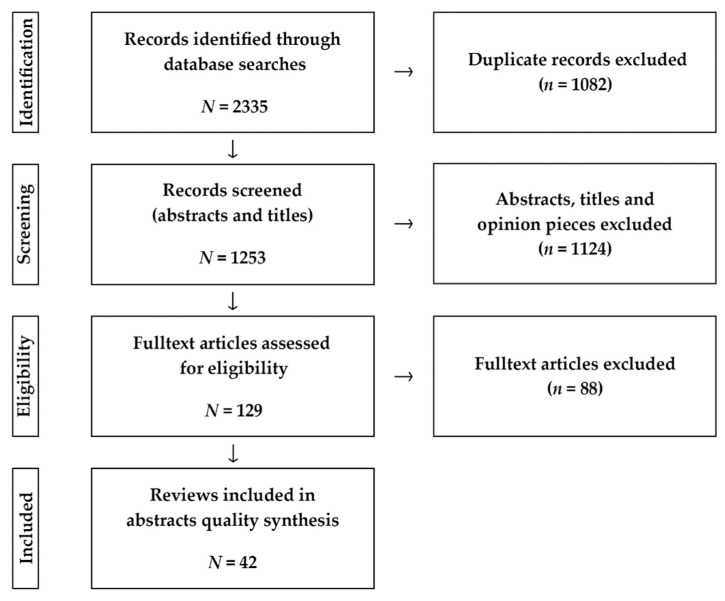
Article selection process.

In order to carefully evaluate the existing literature, this review referred to the selection process and filter criteria of other review articles, and conducted quality analysis on the selected articles. We listed the search strategy and filter criteria and then screened the literature by specification (Figure 1).

Additionally, there are many different definitions of urban green space and mental health [9]. In order to clarify the range of this paper, it is very important to explain the definitions of the two in advance. Urban green space refers to “land that is partly or completely covered with grass, trees, shrubs, or other vegetation” [2], which has the function of improving the urban environment and providing a variety of places for recreation and entertainment. Urban green space also includes places with “natural surfaces” or “natural environments”, such as urban forests and parks [3,8,10]. Similarly, it is not sufficient to simply define mental health from the perspective of psychiatrists, that is, as the absence of mental illness [11,12]. In this paper, mental health is more broadly understood as a normal state of mind. For example, having a better mood and less stress and anxiety can be regarded as having a healthier mental state [13,14,15,16].

### 2.1. Search Strategy and Filter Criteria

We searched Web of Science and Scopus in December 2020. For the purposes of this review, the search terms for urban green space included “green space” OR “open space” OR “urban space” OR “urban forest” OR “forest therapy”. The search terms for mental health included “mental health” OR “psychological relaxation” OR “psychological health”. In order to avoid missing the articles that illustrate the relationship between mental health and urban green space from the perspective of human well-being, the combination term, restore OR restoration AND “human well-being”, was also considered. All these terms were searched in titles, key words, and abstracts. Combinations of the search terms were also run in these databases.

We included (a) empirical studies and research designs from around the world and made no restrictions relating to gender, age, nationality, region, or race, and (b) both descriptive and observational studies were included. Among them, (c) either cross-sectional or longitudinal designs, randomized controlled trials or intervention trials were acceptable. The cross-sectional study is good at identifying and measuring the strength of the relationship between green space and mental health [17]. The longitudinal design can provide a timeline which can satisfy the study requirement relating to causation [18]. (d) The articles must include possible mechanisms or mediators between green space and mental health. (e) Green space was analyzed empirically, either by objective methods based upon geographic information system (GIS) or other available data, or otherwise by subjective methods using standardized questionnaires. (f) The factors measuring mental health included mental state, mood (i.e., pleasure, happiness, depression, stress, anxiety, and other positive or negative emotions), and restoration.

In addition to following the above criteria, there are several points to be clarified during the screening process. After filtering the two databases, we first eliminated duplicate articles. For linguistic reasons, (g) we excluded the studies if they were not written in English.

At the stage of title and abstract filtering, we mainly considered the correlation between the search results and the search terms, as well as whether the research focused on urban green space and health. (h) Some studies were only related to either urban green space or health. These studies were excluded. In addition, (i) we also excluded studies if they described the general benefits of nature. Since our research subjects were urban residents, (j) we excluded articles that were non-urban or non-human studies (e.g., those conducted using mice). In our research, we sought to draw conclusions regarding the path of the impact of green space on mental health, according to the research process. Therefore, (k) we excluded studies if they were reviews, meta-analyses, or qualitative or planning articles. Furthermore, we have selected articles that included heterogeneity analysis, that is, exclude those articles that did not analyze the differences caused by population characteristics or carry out descriptive statistical analysis of the research objects.

In the full-text screening stage, in order to prevent the review from producing biased results, (l) we did not screen the results of the study or exclude those articles showing insignificant mediating variables or negative effects. (m) For articles with similar methods and conclusions, we included only one of them, which was always the most comprehensive or most recently published one. In addition, (n) generalized or mixed studies of green space (e.g., green and blue space) were excluded, because it is hard to judge the contribution of different spaces on resident’s mental health.

### 2.2. Data Extraction

Key data from each selected article was collected and extracted into a complete data collection form in Microsoft Excel. The selection of attributes referred to previous reviews on green space and mental health. [7,16,19]. This form included the publication year, study location, sample characteristics, green space calculations/measures, mental health measures, study design, key findings, and potential mediators. Considering the space limitations and readability of this review, a table presenting the key data is shown in Appendix A.

## 3. Results

Figure 1 shows the process of including or excluding articles from the review. From the databases considered, 2330 articles were identified. After discarding 1082 duplicates and 37 papers in other languages, 1124 were excluded at the title or abstract screening stage, and the full text of 129 was assessed. In total, we found 42 articles that met our criteria and received a high score of quality analysis (Supplement Tables S1 and S2. It should be noted that these 42 articles were our main analysis objects. The other information collected during the process of full-text screening will be presented in following sections.

To date, numerous studies have shown that there is a correlation between green space and mental health, but such a correlation cannot represent causality [20,21]. After the key data were extracted (Appendix A), we summarized and classified the influencing mediators obtained and depicted the main paths from urban green space to residents’ mental health. From the perspective of types of green space [22,23], we sorted the articles in Table 1 according to the mediators. Most mediators of environmental factors and social cohesion are found in neighborhood green spaces. Parks work primarily by promoting outdoor activities. Urban forests focus on subjective perception of sense of belonging and security as a mediator in social cohesion.

**Figure 2:**
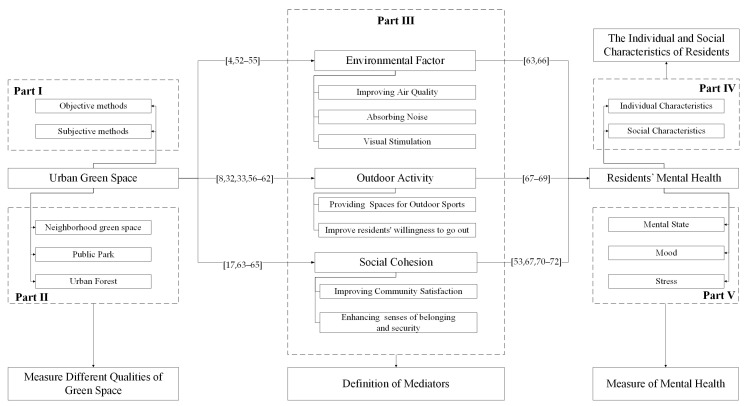
The mediators and influencing paths of urban green space’s impact on residents’ mental health [4,8,17,32,33,44,51,52,53,54,55,56,57,58,59,60,61,62,63,64,65,66,67,68,69,70,71,72]. Notes: (1) Part I and Part II are the classification and measurement of urban green space, respectively, which is defined in Section 2. (2) Part III is the mediators of urban green space’s impact on residents’ mental health, which is the core part of the article. This part will be shown in Section 3.1. (3) Part IV is the heterogeneity characteristics of residents. The heterogenous effect of different mediators on various groups is shown in Section 3.2. (4) Part V is the measurement of mental health, which is presented in Appendix A. (5) Based on part III, the classification and definition of mediators is discussed in Section 4.1. (6) Based on part IV, the individual and group characteristics of residents are discussed in Section 4.2. (7) Based on parts I and II, the classification study and quality analysis of green space will be discussed in Section 4.3. (8) Based on parts III and V, measures of mental health are discussed in Section 4.4 in terms of mediator.

The possible mediators that exist between the two were analyzed, and the possible influencing path was deduced (Figure 2). Figure 2 also summarizes three influencing mediators relating to mental health. Then, different mediators were analyzed and compared from the perspective of heterogeneity.

### 3.1. Impact Mediator

Urban green space affects residents’ mental health. However, how does this effect come about? That is why we are reviewing and considering mediating variables and influencing pathways. The impact of mediators on urban green space and mental health should be a factor, but is one that is not included in the concept of urban green space or mental health. For example, the quality of green space and the time spent in green spaces are measures of green space, and the perception of loneliness and stress are indicators of mental health, which cannot be regarded as the mediators we are discussing. Mediators can be divided into direct and indirect mediators [48,73]. A direct mediator refers to promoting mental health through the characteristics of plants themselves (such as improving air quality, absorbing noise, and visual stimulation), which is summarized as “environmental factors”. Indirect mediators focus on the use of green space [26,31,64]. On the one hand, green space can provide places for outdoor sports and social communication and attract residents to engage in outdoor activities, which is summarized as “outdoor activity” [28,32,33]. On the other hand, from residents’ subjective perception, green space can enhance their senses of belonging and security and their neighborhood satisfaction, which is summarized as “social cohesion” [42,43,74,75]. Hence, from the two perspectives of direct contact and passive attraction, we summarize the mediating factors between green space and mental health into three aspects: environmental factor, outdoor activity, and social cohesion. This corresponds to Part III of Figure 1.

#### 3.1.1. Green Space Affects the Environmental Factor

According to the definition of green space, it is obvious that the addition of green plants can improve the environment. The environmental factors discussed here are mainly considered from the perspective of human beings. From an objective point of view, some of the characteristics of green plants themselves directly affect the residents. On the one hand, green space can reduce physical damage to the residents [26,76]. On the other hand, greenery can increase the visual stimulation of the residents [31,64,77].

First, green space can absorb pollutants from the air and improve air quality. Green space has a significant impact on pollution exposure [51]. Yin and Yuan indicated that increasing green space can mitigate the urban heat island [78], and thus improve air quality [79]. Gascon et al. [27] suggested green space has a potential protective effect on mental health (depression and anxiety) in adults, mediated in part by air pollution and, to a lesser extent, noise exposure. Franklin et al. [26] indicated that people’s exposure to smoke at home and residential exposure to artificial light at night and near-roadway air pollution were associated with increased perceived stress. These connections seem to be partly mitigated by more residential green space. Urban green spaces will reduce residents’ sensitivity to stress [24,42,61]. These results may provide a theoretical basis for green space to reduce environmental pollution and improve residents’ mental health [26].

Second, green space also absorb noise from the environment, thus reducing the stress of living and promoting mental health of residents. Several studies have been conducted to explore this link. The presence of vegetation can also weaken the negative perception of noise, to a certain extent [8]. Plants have a greater ability to maintain attention. This allows residents to better relieve the self-perception of pain and relieve pressure, thus adjusting the psychological state and improving people’s mental health [56,73]. Yang et al. [24] found that high levels of stress affected sleep quality, but the impact of stress was relatively small in neighborhoods with large amounts of green space. In other words, green space can improve sleep quality by absorbing noise.

Furthermore, green space can allow for visual stimulation, which can make people’s minds more relaxed. Horiuchi et al. [31] suggested that visual stimulation, such as viewing a real forest, might produce psychological benefits for human health, compared to not viewing a real forest. This stimulation may be associated with feelings of comfort, which lowers blood pressure, heart rate, and psychological stress. Different green space areas have different decompression effects. Van et al. [64] showed that respondents who lived in neighborhoods with more green space were less affected by stressful life events than those who lived in neighborhoods with less green space. The abovementioned results emphasize that green space has a buffering effect on stress.

#### 3.1.2. Green Space Affects Residents’ Outdoor Activity

Outdoor activities not only include sports but also leisure activities such as walking, social contact, and interaction with residents. It is an indirect mediator, which is a spontaneous activity of residents. It is not entirely dependent on green space, which means that residents can engage in outdoor activities without green space. Green space simply indicates an outdoor space with more vegetation; that is, residents prefer to go to green spaces for outdoor activities [33]. Because of this, residents are more willing to engage in outdoor activities; that is, green space strengthens the motivation of residents to perform outdoor activities.

Green space can provide spaces for outdoor sporting activities and opportunities for physical activity. The duration of time spent engaging in green space has an effect in reducing stress levels [80,81]. A study conducted by Lu [65] in Swedish towns and cities showed that the amount of time residents spent outdoors in green areas is inversely related to their own stress. A brief recreation program in the forest may be effective in reducing the negative symptoms of stress [82]. Whether walking in the suburbs or in the forest, participants felt relaxed physically and psychologically, and this activity had a positive impact [15]. Forest bathing heightened the positive effects and induced a feeling of subjective restoration and vitality [50,83,84]. Furthermore, Brown [85] used a scale to assess the mental and physical health of participants. Compared to the control group, who performed two walks in an urban environment, the self-reported mental health of the natural walk group improved.

However, some studies have suggested that the relationship between green space and physical activity is not significant. While physical activity is higher in greener neighborhoods, this does not fully explain the relationship between green space and mental health [33]. Richardson et al., showed no association was found between green spaces and social contact or physical activity [86]. One possible reason is that people who like to go out tend to have more positive emotions in the first place. Their positive emotions are therefore not directly related to whether they like going to green spaces. On the other hand, green space can promote interaction between people in society. Kruize et al. [32] showed that more time spent outdoors in natural environments is associated with more time engaging in social contact with neighbors and better mental well-being. Brockman et al. [87] showed that several features of the physical environment promoted active play for children, including green spaces in the community.

In addition to physical activities, social activities are also a part of outdoor activities. Social activities can further enhance contact and interaction between residents. More specifically, Dadvand et al. [25] showed that spending more time in green spaces was associated with increased self-satisfaction and social interaction. Social contacts explained more than half of the link between green space use and self-satisfaction [46]. Yao et al. [20] concluded that green space provides a good environment for social activities, increases the possibility of communication between neighbors, and promotes social interaction among residents. Furthermore, social interaction is an essential part of personal life. The health status of people who participate more frequently in social activities is better than that of people who participate less frequently [53]. The health status of social people is interrelated to the interaction promoted by urban green space [35].

#### 3.1.3. Green Space Affects Residents’ Social Cohesion

Social cohesion has been defined in many ways. Unlike social activity in outdoor activity, social cohesion starts from human perception. In this review, we see social cohesion as residents’ senses of belonging and security, and their neighborhood satisfaction [42,43,74,75].

Green space affects adults’ perceptions of loneliness, security, and happiness. Research by Maas et al. [88] showed that even if adults did not have much contact with the people around them, they were less lonely as long as they lived in an environment with a high proportion of green spaces (including parks, farmland, and forests). Greiner et al. [53] pointed out that open green space is an important place for urban social interaction and demonstrated the positive correlation between social participation and mental health through a questionnaire survey of residents. Urban parks in green spaces, as places of social interaction, could enhance people’s sense of security and belonging, and the wide visual space created by green spaces might also reduce the incidence of crime [43].

Green space can improve residents’ environmental satisfaction. Empirical research shows that residents’ subjective perception of neighborhood characteristics has an impact on residents’ neighborhood satisfaction [89]. By studying neighborhood satisfaction, we can measure residents’ quality of life to a certain extent and infer the mental health of residents [70]. Kaplan [90] found that viewing natural elements from a window is conducive to improving residents’ satisfaction with green space environments and various aspects of happiness. Fried [70] proved that proximity to nature is the most powerful single predictor of neighborhood satisfaction, in the means that green space can greatly enhance social cohesion. Hur [91] used GIS and Landsat satellite images to measure the characteristics of green space in Franklin County in central Ohio in the United States. Through path analysis, the study found that there is a correlation between the characteristics of a neighborhood environment (green space) and residents’ subjective and perceived neighborhood satisfaction.

Furthermore, residents’ perceived neighborhood satisfaction links environmental characteristics with self-assessed mental health, that is, neighborhood satisfaction is the link between green neighborhood space and happiness [92]. Leslie et al. [52] confirmed that the environmental characteristics of residents’ residences, such as the aesthetics and greening, the diversity of mixed use of land, and other factors, are positively related to neighborhood satisfaction. These factors affect residents’ mental health, that is, many aspects of neighborhood satisfaction are related to self-reported mental health. Choumert et al. [93] confirmed that people who are dissatisfied with their surrounding environment due to a lack of green space or for other reasons have worse mental health than those who love their surrounding environment. Nielsen et al. [63] confirmed that the closer the residence is to green space, the lower the stress felt by residents.

It is worth noting that such experimental studies can only observe a significant correlation between the environmental characteristics of a specific green space and social cohesion through the joint significance test [52], that is, social cohesion is a good mediator between perceived green environmental characteristics and mental health. The argument relating to causality is yet to be perfected.

### 3.2. Heterogenous Effect in Different Mediators

The impact of green space has a heterogeneous effect, i.e., green space affects people in different ways. It has been suggested that certain residents, such as children, the elderly, and women, may benefit more from the presence of neighborhood green space than others [94]. The reason is that different mediators work on people of different ages in various ways and have various effects. For example, green space mainly promotes the mental health of children, adults, and the elderly by enhancing peer communication, relieving stress, and increasing outdoor activities, respectively. The connotation of heterogeneity is very diverse. In addition to age, gender, income, education level, and family structure can also be considered. Additionally, subgroups such as pregnant women or people with disabilities or allergies, and characteristics of the city where they live, also need to be taken into account. In the following, we describe the influence of different mediators on these heterogeneities. This corresponds to Part IV of Figure 1.

#### 3.2.1. The Heterogenous Effect of Environmental Factor

Environmental factors refer to the impact of green space on residents’ mental health by reducing physical damage and increasing visual benefits. From the perspective of age, this effect is particularly pronounced for children with cognitive immaturity. Dadvand et al. [25] showed that there was a beneficial relationship between green space exposure and the cognitive development of schoolchildren, which was mediated to some extent by the reduction in air pollution exposure. For the elderly, those living in low-quality housing have a stronger demand for green space. They are more likely be affected by light, smell, greenness, temperature, and humidity [95].

For people who are sedentary or have limited mobility, the environmental factor of green space can be achieved by appreciated green areas through the window. Furthermore, it is easier to make small repairs through the window [43]. This visual stimulation had a stronger effect on mental health than activity in green spaces [30]. Reducing pollution through green space can reduce the risk of depression for residents. However, for people with allergies, green space actually reduces the effects on mental health by improving air quality [96]. From urban density type, increasing green space will aid the residents’ mental health in compact urban areas by reducing urban density [79,97]. For poor regions, neighborhood green space may promote emotional well-being in poor urban children in early childhood through visual stimulation [98].

#### 3.2.2. The Heterogenous Effect of Outdoor Activity

For the elderly, time spent in parks is associated with mental health, and physical activity also helps promote mental health. Older people’s own physical conditions can also affect their tendency to go outdoors. Elderly people with cardiovascular disease are more likely to go to a park, while those with hypertension are less likely [36]. In consideration of their physical condition, there is another prerequisite for the elderly to engage in outdoor exercise, that is, the perception of environmental safety. However, this point is slightly different in the case of women. The precondition for women to choose outdoor sports is the consideration of green area security. Physical activity near parks is associated with good mental health, but only for those who do not care about park crime [35]. Green spaces with limited horizons, such as parks, can deepen women’s sense of insecurity.

In terms of subgroups, green space encourages pregnant women to engage in outdoor leisure activities to a certain extent [99]. This is a small but significant mediator. People with dogs have more opportunities for outdoor exercise than other residents and can make better use of green space.

Residents in cities with high levels of urbanization spend more time sitting and less time on sightseeing and outdoor activities, thus weakening the mediating effect of outdoor activities [100]. In high-density cities, outdoor exercise plays a significant mediating role [48]. There are also opinions that cities with high urbanization level have more leisure green space, better accessibility, and more opportunities for outdoor sports than rural farmland, so outdoor sports have a significant mediating effect.

#### 3.2.3. The Heterogenous Effect of Social Cohesion

Green space enhances children’s interaction with nature and affects their cross-cultural communication and growth [101,102]. Frances et al. [71] found that the interaction between natural environments and animals is extremely important for children’s growth. Echeverria et al. [74] confirmed that urban green space, such as parks and playgrounds, can significantly promote cross-cultural contact and friendship between children. Sedentarism can lead to poor mental health in children, while spending time in green spaces can improve this situation. Andrusaityte et al. [34] showed that residential greening and time spent in parks are positively correlated with a reduction in children’s general and mental health risks. An increase in time spent playing and interacting can allow children to resolve emotional problems and establish peer relationships, and it can increase their concentration [44].

Green space affects adults’ perceptions of loneliness, security, and happiness. Research by Maas et al. [88] showed that even if adults did not have much contact with the people around them, they were less lonely as long as they lived in an environment with a high proportion of green spaces (including parks, farmland, and forests). Greiner et al. [53] confirmed that urban parks in green spaces, as places of social interaction, could enhance people’s sense of security and belonging, and the wide visual space created by green spaces might also reduce the incidence of crime. Dadvand et al. [103] observed that some signs of underlying age and sex differences appeared to be more relevant to male participants and people younger than 65 years of age in these mediating roles related to mental health status and perceived social support. In addition to the benefits of green space, women had some concerns about the safety of green space. The negative effects weaken the positive effects of social cohesion to a certain extent [99]. This may be the reason why social cohesion is not significant for female residents.

## 4. Discussion

Through an analysis of a series of previous studies on green space and mental health, it is not difficult to find that studies of the same population often draw different conclusions, and the significance of each mediator is not the same in different studies. A green space is a geographical system with rich functions and a complex structure. Each country has different characteristics in terms of the climate, status of development, and living conditions. For example, in countries with poor sanitation, living in green spaces may be detrimental to mental health because such areas have a higher risk of infectious diseases [104]. On the other hand, in cities in low- and middle-income countries which are developing faster than high-income countries, mental health problems are almost ignored [4,101]. Apart from these, the various urban green space rates, tree species mixes, etc., are different, so their ecological health functions and impact on the health of urban residents are also different [105]. Consequently, researchers need to substantiate and clarify what exactly is the mediator between green space and mental health. Furthermore, it is necessary to fully consider differences in the heterogeneity of residents, green space quality, and measures of mental health.

### 4.1. Definition of Mediators

Based on Part IV of Figure 1, we summarized the mediating factors between green space and mental health into three aspects. However, the definition of mediators is not uniform. There is a lot of good research going on, and most articles consider the role of mediators. The way they look for mediators is to refer to historical studies, propose mediation hypotheses, and verify the significance of the mediation through models. However, this approach is not able to ensure that these mediators are mutually exclusive and collectively exhaustive.

The problem is that the factors identified in this way are not necessarily mediations, which, in many literatures, overlap with the concepts of green space and mental health. For example, the concept of loneliness may be included in the measurement of mental health [43]. It is a dimension to describe mental health. The same goes for stress [42]. Even different studies disagree on whether stress is a mental health issue. Similarly, mediation overlaps with green space. Some researchers take the use time of green space as a mediating variable, which seems to be a measure of the use rate of green space (many related papers take it as an independent variable). Additionally, if we continue to ask why the more time we spend in green space the healthier our psychology, we still need to continue to solve the mediation problem. Some studies contend that greening quality is also a possible mediating variable. However, since it is not related to any greenbelt index, and it is not easy to measure.

From our point of view, the impact of mediators on urban green space and mental health should be a factor that is not included in the concept of urban green space and mental health. Yet, the study of mediators is not over. It needs more researchers to pay attention to this problem and more evidence to further support and improve relevant theories.

### 4.2. The Individual and Social Characteristics of Residents

There are many aspects of heterogeneity. Various influencing factors, such as individual characteristics and social characteristics of residents, should be considered comprehensively to reduce random errors to the greatest extent, so as to clarify the mediator of green space on mental health. In the future, the correlation between urban green space and residents’ mental health should be demonstrated in a broader space–time scope. Researchers should try to avoid the existence of confounding factors in sample screening, and long-term follow-up observations should be conducted on participants’ mental health to improve the effectiveness of the results. Therefore, we need to fully consider the individual characteristics and social characteristics of the residents in the research process to ensure the accurate analysis of how the mediator works. This corresponds to Part IV of Figure 1.

The individual characteristics of the residents need to be included in the category of research variable control. In most studies, the analysis’ object is an individual. Only in a few “time–activity” detection studies has a specific area been taken as the research object [106]. Therefore, the research sample can only exclude some medical prerequisites, and there are always uncontrollable potential confounding factors between subjects, such as individual differences (health prerequisites, mental conditions, etc.) [107].

The social characteristics of the sample population need to be included in the category of research variable control. Some studies have found that the health benefits of green spaces can be modified by variables such as education level and socioeconomic status [4,98]. For example, a British study found that the risk of emotional problems among poor children aged 3–5 was related to the surrounding green environment, but not among children from a higher social status [98]. For example, people with different levels of education perceive the effect of green space differently. Pun et al. indicated that there was a significant negative correlation between green space and perceived stress in highly educated people. Because these people spend more time near the home, they use and interact with their surroundings more frequently [108]. As mentioned above, people with a lower socioeconomic status seem to benefit more from green space, and few studies have focused on the impact of urban nature on vulnerable people, that is, the issue of “environmental injustice” [107].

Stratified analysis can be conducted according to social class, education level, age, and gender. These factors may change the direction and extent of the impact of green space on mental health, which means different mediators and influencing mechanisms.

### 4.3. Types and Qualities of Green Space

Urban green space includes neighborhood green space, urban forests, and parks, which corresponds to Part IV of Figure 1. There is currently no standardized approach to define green space, specifically, to define what we actually mean by surrounding greenness or exposure or access to green space [109]. This relates to the heterogeneity regarding green space assessment among different studies. Few studies have examined the association between mental health and the type and quality of green spaces, and only some researchers have studied the impact of environmental conditions on artificial and natural green spaces and the impact of improved and unimproved green spaces on participants’ mental health. For example, Butryn et al. [92] measured the emotional and sensory states of female long-distance runners, before and after running four miles on a natural or man-made urban route. The results showed that people’s emotional and sensory states were improved in both cases. Olszewska-Guizzo et al. used type of urban green space as a substitute for quality of green space. Specifically, parks were regarded as green spaces of higher quality than neighborhood green spaces [10].

The quality of green space should not be discussed in general in the research on green space and mental health. Instead, the different dimensions of green space quality should be explored according to the different tendencies and emphases of different mediators. The green space index (GSI) has been used by Occidental countries in recent years to quantitatively evaluate green infrastructure in designated sites. By superimposing the different weights of green space types, which have different ecological benefits, and by comparing them with the minimum value of the set index, a quantitative reference space can be obtained. Among them, ecological service, infrastructure spatial allocation, and maintenance of green space correspond to environmental factor, outdoor activity, and social cohesion, respectively.

Nowadays, cities around the world that have implemented the GSI have received favorable feedback from city managers, project builders, and the general public. Taking the Berlin habitat index as an example, relevant surveys have shown that, since its implementation, urban green infrastructure has achieved remarkable results in terms of regulating the urban ecological environment, improving the environmental quality of residents, and promoting the health of residents [110].

In a word, measuring the type and quality of green space from the perspective of mediators is more conducive to exerting its benefits, thus promoting the mental health of residents. The practice of urban green space-related policies has been widely carried out across the world, and the psychological health benefits of residents are relatively significant. There is an urgent need to fully consider mediators to distinguish types of urban green space and measure green space quality and to study the positive effects of urban green space on residents’ mental health from the perspective of type and quality of urban green space. The purpose is to ensure the comparability of related researches on urban green space.

### 4.4. Measure of Mental Health

Based on the measurement methods of mental health, empirical research was conducted on urban green space and the mental health of residents. With the help of specific measurement tools, the relationship between these psychological factors and environmental factor, outdoor activity, and social cohesion was further analyzed. Such methods are more diverse, focusing on the use of observation methods and interviews, while preferences and other behavioral social survey methods are based on scales. The measures of mental health in the selected literature are shown in Appendix A. Mental health measures are mainly divided into three categories, including mental state measures, mood measures, and restoration measures. These three are correlated with the mediators, which is based on Parts III and V of Figure 1.

Environmental factors as a mediator mainly affect mental health from the level of recovery, such as improving air quality, reducing ambient noise, and increasing visual stimulation. Therefore, the mental health under this mediator is mainly measured by restoration. It refers to the relief of stress and psychological relaxation. The restorative outcome scale (ROS) is used to assess human recovery of forest environments [82]. The perceived restorativeness scale (PRS) measures how much mental alertness is restored in a given environment [49]. The Kessler Psychological Distress Scale (K10) measures symptoms of psychological distress experienced by subjects [38]. Fan et al. [13] measured stress using the Perceived Stress Scale (PSS).

Outdoor sports often bring about interaction between residents and people or the environment. The effects on mental health tend to be direct in mood. Positive emotions are part of mental health. The most commonly used are the Center for Epidemiological Studies Depression Scale (CES-D), the Profile of Mood States (POMS), and the Depression Anxiety Stress Scale-21 (DASS-21). The Positive and Negative Affect Schedule (PANAS) is also used to evaluate the positive and negative feelings of participants, and it has already been applied in many studies [15,49,82].

Social cohesion refers more to the residents’ subjective feelings about their living environment, so it often corresponds directly to the mental state scale. Some studies use the General Health Questionnaire (GHQ) [86,111] to measure the effectiveness of exposure to quiet and spacious green spaces in reducing the risk of poor mental health in women, and some studies use the Mental Health Scale (MHI) to measure mental health [42,112]. The short form health survey (SF) is also a valid instrument for measuring mental health [33,52,86].

Of course, this correspondence is not absolute. Some studies measure the impact of environmental factors and outdoor activities on mental health directly through mood. By recording the electroencephalography (EEG) signals of participants, Olszewska-Guizzo et al. [10] found that participants in green spaces produced higher frontal alpha asymmetry (FAA) values, which are generally associated with subjective motivation and positive emotions. By assessing children’s internalization and externalization ability (basc-2), we can assess the general mood and behavior symptoms of adolescents [25].

## 5. Implications for Green Space Planning

Green space plays an increasingly significant role in residents’ life. More and more urban policy makers are including green space in urban planning and considering the coordination between green space and building in order to maximize the health benefits of green space. The planning and design of urban green infrastructure is the mainstream policy practice in urban green space construction. According to David Ross, an American Landscape Architecture Planning scholar, green infrastructure is an internally connected green space ecological network which is formed by combining the natural environment and artificial environment [93]. This network can perform a series of urban ecosystem functions and improve people’s health, especially mental health, by creating more green spaces. Community is the basic unit of social governance. To solve the problem of community green space is to meet residents’ demands for green space from the micro perspective. The green space of the community should be planned according to the environmental characteristics of the community in a people-oriented way [113]. For this reason, and in consideration of green space quality and heterogeneous demand, the policy implications of green space planning are put forward at different levels of mediation by drawing on the experience of other countries.

### 5.1. Implications on Environmental Factor

The mediating effect of green spaces’ environmental factors is the direct harm reduction and the physical gain to residents. Green space, such as the plants in the streets and office buildings, works by reducing air pollution and environmental noise and increasing green visual stimulation. Therefore, urban planning needs to ensure that there is enough green space between buildings and roads.

To be specific, the construction of green and gray infrastructure should be coordinated. Grey infrastructure is the traditional municipal public infrastructure which has a single function, such as roads and bridges. Green infrastructure is a green space system, which should be connected with grey infrastructure. The accelerated development of urbanization leads to green infrastructure not being able to play its role in promoting health independently, which creates the necessity for networked support of gray infrastructure. It is necessary for densely urbanized regions to apply a more environment and ecosystem friendly planning approach and system [97]. Urban administrators need to balance the two and promote the construction of green infrastructure to the maximum extent, while improving grey infrastructure. The practice in Cleveland, Ohio in the United States is a good example. The government advocated that relevant departments give priority to the development of green building standards when revising local gray infrastructure regulations, such as the construction of ecological botanical gardens and the expansion of residents’ green activity spaces, which provided mental health benefits to local residents to varying degrees.

### 5.2. Implications on Outdoor Activity

The mediating effect of outdoor activities is that green space strengthens residents’ behavior. In order to make it work, we must first ensure that residents have green space, want to have green space, and have access to green space. This involves three aspects of availability, security, and accessibility. In addition to the guarantee of the quantity of green space, we should also consider the improvement of quality. It is important to add facilities that enhance the quality of sports or social activities.

On the one hand, full consideration should be given to special groups, such as the disabled, the elderly, children, etc. Combined with heterogeneity, the different demands for green space should be fully considered from the perspective of all age groups, so that the green space can be reasonably allocated to all residents. For example, for the elderly and children, special activity areas can be set up, and corresponding entertainment facilities, fitness facilities, and rest seats can be added. For the disabled, sloping passageways can be designed to ensure green space accessibility. In addition, the open vision of green space should be fully considered in order to improve the sense of security of residents’ while they undertake activities in the green space.

On the other hand, regarding the quality of the green space, we should not only consider whether the green rate is up to the standard, but also fully consider the accessibility and convenience in terms of actual use. First of all, community green space is the most frequently used activity space for community residents. In order to ensure its use, it is necessary to make public green space more attractive to residents as much as possible. Therefore, the design of community public green space should be more beautiful and interesting in order to increase the frequency of residents using it. Secondly, the greening design should fully integrate the spatial structure of the community and the behavioral habits of residents in order to ensure the availability and accessibility of the green space and, thus, minimize any negative effects on the convenience of residents’ life.

### 5.3. Implications on Social Cohesion

The mediating effect of community cohesion is to increase residents’ sense of belonging and satisfaction with the surrounding environment. Policy makers need to organically combine the urban production environment and green space. Meanwhile, as a kind of environmental resource, monitoring and maintenance of green space also needs to be considered.

The connection between green infrastructure and the local environment is deep. In the planning and design of green space, we should pay attention to the regional concept, preserve the natural landscape, and reduce the damage to the original ecological landscape. The transition between the buildings and the surrounding green space should be considered at the same time. For example, with the help of the tributaries of urban rivers, the continuity principle was adopted to build an ecological park in Louisville, Kentucky, USA. The project of Queen’s Square Park in the United States involved the use of a large number of green plants to green the dangerous intersection, which is integrated with the characteristics of the natural area. The project not only ensured road safety, but also improved the health and well-being of residents. In Maryland, USA, rain gardens provided extended green spaces for urban residents. Because of the low costs of construction and maintenance and high health benefits, these gardens were widely adopted around the world.

In addition, community green space should be constantly transformed and maintained. Managers should combine practical experience and pay continuous attention to the green space of the community. According to the change in residents’ demands, the green space and related facilities should be restored and updated regularly. On the basis of reasonable planning, the management of community green space should be strengthened. For example, private occupation of public green space and malicious damage to the green environment by some residents should be prevented.

## 6. Conclusions

At present, fruitful achievements have been made in the research on green space and mental health. Through the above review, the path of the impact of green space on residents’ mental health is fully discussed and analyzed. We summarize the current mediators and identify the impact paths of different mediators. Furthermore, this article specifically analyzes the heterogeneous effects of the above-mentioned influences, considering not only different types of green spaces, but also residents with different socioeconomic characteristics. From the perspective of direct contact, the environmental factor is considered as the main mediator, which includes improving air quality, absorbing noise, and visual stimulation. It mainly works through the neighborhood green space. It can be helpful for adults to reduce stress and improve sleep quality, which in turn improves their mental health. From the perspective of passive attraction, this can be divided into outdoor activity and social cohesion. Parks and urban forests provide venues for residents to engage in outdoor activities and communication, and make them mentally healthier. Children and the elderly often benefit from this impact pathway. A greener environment can improve residents’ sense of social satisfaction and happiness in life. These results are more consistent in dense cities. These findings should make an important contribution to the field of causality analysis between urban green space and residents’ mental health, as well as demand-oriented urban green space planning and management.

In addition, there are limitations to the study. When analyzing the heterogeneity, we cannot guarantee that every mediator contains all the dimensions of heterogeneity due to the limited search results. In future studies on green space, it will be necessary to fully consider the impact of heterogeneity, including not only the individual, but also the social characteristics of samples, and to adopt a relatively uniform standard to measure green space. Detailed and comprehensive research should also be carried out on the mechanism between green space and mental health, including a study of the mixed effects of the mediators.

## Figures and Tables

**Table 1:** Summary of selected articles based on types of green space and mediators.

	Neighborhood Green Space	Park	Urban Forest	Other or Unclassified Green Space
**Environmental Factor**				
Improve air quality	[24,25,26,27]			
Absorbing noise	[24,27]			
Visual stimulation	[28,29,30]		[31]	
**Outdoor Activity**				
Physical activity	[29,31,32]	[32,33,34,35,36,37,38,39,40,41]	[15]	[32,42,43]
Social activity	[32,44]	[32,45,46]	[46,47]	[43]
**Social Cohesion**				
Neighborhood satisfaction	[10,17,28]	[46]		
Sense of belonging and security	[17,30,48]	[34]	[46,49,50]	[42,43]

## Supplementary Materials

The following are available online at https://www.mdpi.com/article/10.3390/ijerph182211746/s1, Table S1: Quality assessment tool, Table S2: Quality assessment score.

## Appendix A

**Table A1:** Main characteristics and results of the studies on green space and mental health.

No.	Publication Year	Study Location	Sample Characteristics	Green Space Calculation/Measures	Study Design	Key Findings	Potential Mediators
[5]	2013	England	5000 households and 10,000 individual adults	The Generalized Land Use Database (GLUD) classifies land use at high geographical resolution across England and has been applied to 32,482 lower-layer super output areas (LSOAs)	Panel data analysis	A greater amount of green space is associated with less mental stress and greater happiness.	Stress and neighborhood satisfaction
[10]	2020	Singapore	22 healthy volunteers (13 females; mean age = 32.9, standard deviation = 12.7)	Contemplative landscape score	Electroencephalography (EEG) technology was used to test the changes in a busy urban street, an urban park, and a neighborhood green space to test the mood swings of participants.	In green space, participants’ Frontal alpha asymmetry (FFA) is more significant, which means that they have more positive emotions.	Positive emotion
[52]	2008	Adelaide, Australia	2194 residents aged between 20 and 65	Neighborhood environment walkability scale (NEWS–AU)	Principal components analysis with oblique rotation was conducted to identify summary measures of neighborhood satisfaction.	Neighborhood satisfaction may mediate the association between perceived environmental characteristics and measures of mental health in adults.	Neighborhood satisfaction
[24]	2020	Hong Kong, China	608 pedestrians aged 20 years or over	Normalized difference vegetation index (NDVI)	Multinomial logistic regression models were applied to assess the effects of green space on sleep quality and perceived stress.	High levels of stress affect sleep quality, but the effect is relatively small in neighborhoods with a high amount of green space.	Relief of stress
[32]	2020	European cities	3947 adults aged 18–75 years	GIS-derived measures and NDVI	A cross-sectional design was used.	Physical activity, a higher frequency of social contact with neighbors, and better mental well-being	Physical activity and communication with the neighborhood
[33]	2013	New Zealand	8157 adults aged 15 years or over	Green space quartiles	Cross-sectional analysis of anonymous individual health survey responses was conducted.	Although physical activity is higher in greener neighborhoods, it does not fully explain the relationship between green space and mental health.	Physical activity
[86]	2015	Catalonia (Spain)	8793 adults	Indicators of surrounding greenness and access to natural outdoor environments within 300 m of the residence	Cross-sectional analysis was conducted by using logistic regression and negative binominal models.	Instead of physical activity and social support, restoration and stress reduction could be alternative pathways that underlie the associations between green space and mental health.	Physical activity and social support
[28]	2011	Ghent, Belgium	Two inner-city neighborhoods that differ objectively in greenery, with 300 residential households per neighborhood	GIS	Ward’s method of hierarchical clustering was utilized.	Stress is significantly correlated with community satisfaction and happiness, but there is no significant difference in the perception of stress between two communities with different amounts of green space.	Stress and neighborhood satisfaction
[114]	2004	Hamilton, Ontario, Canada	1504 adults aged 18 years and older residing in four contrasting neighborhoods	Subjective experience of residents	Cross-sectional survey data were analyzed in small neighborhoods.	The influence of the physical environment, such as green space, on neighborhood satisfaction is much higher than that of the social environment; people are more satisfied with communities with more green space, and thus are happier.	Neighborhood satisfaction
[34]	2020	Kaunas city, Lithuania	1489 4–6-year-old children	Normalized difference vegetation index and time spent in a park	A cross-sectional study was conducted using multivariate logistic regression models.	Residential greening and time spent in parks are consistently positively associated with a reduction in children’s general and mental health risks, and spending time in parks could ameliorate the effects of sedentarism.	Physical activity
[44]	2014	Barcelona, Spain	2011 schoolchildren (7–10 years of age)	Normalized difference vegetation index and proximity to green space	A cross-sectional study that applied quasi-Poisson mixed-effects models	Green space increases the amount of play time and interaction, thus solving emotional problems and peer relationships and increasing children’s concentration levels.	Physical activity and peer relationships
[35]	2020	New York, United States of America	3652 residents aged 18 or older	Self-reported time to walk to the nearest park from home	Multiple regression with bootstrap-generated 95% bias-corrected confidence intervals (BC CIs) was used.	Physical activity near parks is indirectly associated with fewer days of poor mental health, but only for those who do not care about park crime.	Physical activity
[36]	2019	Bandar Abbas, Iran	1965 elderly people (65 years old or above)	Level of park activity	A cross-sectional field survey was conducted from a population-based randomized sample of elderly people.	Older people’s own physical condition can also affect their tendency to go out; people with cardiovascular disease are more likely to go to the park, while those with high blood stress are less likely.	Physical activity
[110]	2013	Four Dutch cities (Utrecht, Rotterdam, Arnhem, and Den Bosch)	1641 residents	Subjective description	Multilevel analysis was conducted to investigate the mechanisms behind the relationship between urban greenery and mental health.	The contribution of green activity is often not significant; there is a possibility that the effect of green activity is mediated by stress and social cohesion, rather than that it has a direct health effect.	Stress and social cohesion
[37]	2013	Edinburgh, Scotland	12 students from Edinburgh University	Subjective judgment	Using the Emotiv EPOC (a low-cost mobile Electroencephalography recorder), participants took part in a 25-min walk through three different areas of Edinburgh and recorded their emotions.	People have lower frustration, engagement, and arousal levels and higher meditation levels when moving into green spaces, as well as higher engagement when moving out of them.	Environmental factor
[98]	2014	England	6384 children (aged 3, 5, and 7)	The percentage of green space within a standard small area	The Millennium Cohort Study (a longitudinal survey)	Poor children in urban neighborhoods with more greenery have fewer emotional problems from age 3–5 than their counterparts in less green neighborhoods.	Emotional well-being
[15]	2020	Poland	75 young adult Poles studying in the largest Polish agglomeration, Warsaw	The green ratio analysis carried out in the Promovolt application for the presented photographs	The physiological and psychological condition of the participants was measured in rooms, before the walk and just after its end. Measurements of pulse and blood pressure of all participants in the study were performed at the same time.	Both walking in the suburbs and in the forest with fall scenery have a positive effect on the physiological and psychological relaxation of participants.	Physical activity
[31]	2014	Japan	The subjects were 15 healthy volunteers (11 men and four women) with a mean ± SD age of 36 ± 8 years.	The viewing of the forest (Forest condition) and the non-viewing of the forest (Enclosed condition)	The physiological and psychological responses of each subject were measured for both the Forest and Enclosed conditions. The subject’s blood pressure variables, saliva amylase, and profile of mood states scores were evaluated before and after both conditions.	Visual stimulation might be required for and accentuate psychological benefits in human health compared to not viewing a real forest, while similar effects on blood pressure and heart rate variables may occur either with forest condition or without enclosed condition viewing a real forest.	Visual stimulation
[82]	2018	East-Central Europe	21 young Polish adults	Map provided by F. Ordon, the meteorological station in Olsztyn–Mazury, the “Light Meter”	A pre-test–post-test design with a short, one-day intervention of the forest recreation program was applied. The participants’ psychological and physiological responses were measured indoors on the day before forest recreation, and then under field conditions on the next day, directly after the forest recreation.	The short forest recreation program may be effective in reducing negative symptoms of stress.	Outdoor sport
[49]	2019	Japan	46 young male undergraduate and graduate university students	Forest Site	A short-term experiment was conducted using the same method in both environmental settings. We then analyzed the intrinsic restorative properties and the restorative effects of the settings and referred to prior research to determine the restorative effects.	The forest setting was a restorative environment with a higher restorative effect than the urban setting but the influence of individual traits was small; distancing (Stress coping), psychological health, and satisfaction with living environment were likely important indicators that are related to the restorative effects in the forest setting.	Environmental factor and neighborhood satisfaction
[50]	2014	Japan	11 or 12 male university students (45 in total) participated as respondents	Four forest environments (located near the towns of Yoshino, Akiota, and Kamiichi and the city of Oita)	Each respondent walked individually around the area during a 15-min “walking” session before noon. They also sat on chairs and viewed the scenery individually during a 15-min “viewing” session in the afternoon after a lunch break.	Forest bathing heightened positive affect and induced a feeling of subjective restoration and vitality.	Outdoor sport
[25]	2015	Barcelona, Spain	2623 schoolchildren without special needs in the second to fourth grades (7–10 years old)	High-resolution (5 m × 5 m) satellite data on greenness (normalized difference vegetation index)	From January 2012 to March 2013, children were evaluated every 3 months over four repeated visits by using computerized tests in sessions lasting 40 min in length.	An improvement in cognitive development associated with surrounding greenness, particularly with greenness at schools. This association was partly mediated by reductions in air pollution.	Air pollution
[26]	2020	Southern California, United States of America	2290 Southern California Children participants	Green space from satellite observations of the enhanced vegetation index were linked to each participant’s geocoded residence	In this cohort study, a total of 2290 Southern California Children’s Health Study participants residing in 8 densely populated urban communities responded to detailed questionnaires.	People’s exposure to smoke at home in addition to residential exposure to artificial light at night and near-roadway air pollution were associated with increased perceived stress. These associations appeared to be partially mitigated by more residential green space.	Air pollution
[108]	2018	The United States	Older adults (n = 4118; aged 57– 85 years)	The normalized difference vegetation Index at 250 m resolution, as well as a buffer of 1000 m	Longitudinal analyses to assess the associations between greenness and mental health upon adjusting for confounders (e.g., education), and to examine potential mediation and effect modification.	The association between green space and depressive symptoms was significant for active people. Only in physically active individuals was greater green associated with improved anxiety and depression symptoms.	Physical activity
[45]	2019	Hong Kong and Tainan, China	326 older adults	Spatial distribution and accessibility, characteristics of plants and urban green spaces	Two rounds of questionnaires were conducted, with the first round as a pilot study and the second round as in-depth interviewing involving planning and design aspects.	A longer urban green space visit duration creates positive impacts on older adults’ mental health and social functioning. Nicer-looking urban green spaces were considered safer. Older adults preferred to have a greater number of flowers in the urban green space.	Visual stimulation
[39]	2019	Korea	11408 participants aged 65 years and older	Using the proportion of urban green area per administrative area derived from Community Health Survey data to assess the degree of exposure to green space.	A binary logistic regression analysis, with reported symptoms of depression and stress levels as response variables for mental health indicators	The prevalence of these mental health issues generally decreased in relation to the ratio of green space of an area. The higher the rate of greenery in a city, the less stress and fewer symptoms of depression reported among its elderly residents.	Environmental factor
[115]	2014	Plovdiv, Bulgaria	97 elderly adults	Visit specific park (Tzar Simeon Garden)	Hierarchical multiple regression model	The combination of physical activity and natural surroundings has additive antianxiety effects through psychological mechanisms or through better physical fitness and less worry about illness.	Physical activity
[38]	2013	New South Wales, Australia	267,102 aged 45 to 106 years (mean age = 62.8, standard deviation = 11.2)	Using information extracted from ‘meshblocks’ (Australian Bureau of Statistics, 2005).	Loglikelihood ratio test	The link between mental health and greener surroundings as we get older may be increasingly dependent upon our ability to maintain regularly active lifestyles.	Physical activity
[116]	2015	Cambridgeshire, Nottingham, Newcastle and Oxford, England	2424 people aged 74 and over	The percentage of green space and private gardens in each LSOA based on the UK Generalised Land Use 2001 Dataset	Two-level multilevel logistic regression	A high exposure to natural environments (green space and gardens) in communities was associated with fewer mental disorders among older people.	Environmental factor
[40]	2019	Shanghai, China	257 people aged 60 or older without difficulty walking use walking aids;	Selecting some parks based on criteria	Latent class analysis (LCA) was used to detect groups of senior park users with different patterns of behavior in the parks and to understand the groups’ characteristics.	Affective states (i.e., anxiety depression, relaxation, contention) were enhanced after park visits for all subtypes. However, the active park lingerer displayed significantly higher levels of relaxation, compared to the active walker and the passive scanner.	Outdoor sport
[43]	2019	Four European cities: Barcelona (Spain), Kaunas (Lithuania), Doetinchem (the Netherlands), and Stoke-on-Trent (the United Kingdom)	3948 nonhospitalized adults aged 18 to 75 years,	Time spent visiting green space	Physical activity was assessed by the short questionnaire to assess health-enhancing physical activity. To measure social cohesion, the social cohesion and trust scale was used.	Visiting green spaces promotes physical activity, especially during leisure time, and mitigates feelings of loneliness. The effect of green spaces mitigating feelings of loneliness is more important than promoting physical activity as far as mental health is concerned.	Physical activity and social cohesion
[46]	2019	Iran	10,856 adolescents (10–18 years old)	Time spent in green spaces (separately for parks, forests and private gardens)	Logistic mixed effects models with recruitment centre as the random effect were developed to estimate associations adjusted for relevant covariates.	More time spent in green spaces was associated with improved self-satisfaction and social contacts. Social contacts could explain more than half of the association between green spaces use and self-satisfaction.	Social contacts
[41]	2018	Aydın, Turkey	420 respondents, 50.5% (212) were male and 49.5% (208) were female.	Time using green space for physical activity	Multivariate linear regression analysis	Nearest distance to urban green space and quality of urban green space (i.e., maintenance and cleanliness) were associated with increased frequency of physical activity. Large and open/visible urban green space were associated with better physical health.	Physical activity
[29]	2019	Rochester, the United States	142 patients from two cardiac rehabilitation sites	A manual (study-specific) geographical information system (GIS)-based method, the normalized difference vegetation Index (NDVI) and self-reported quantity of green space near the home	Poisson regressions with counts of the dichotomous outcomes for depression, stress, and anxiety.	Increased accessible green space near the home may improve depression and promote recovery in this population. This may be due to physical activity in this space.	Perceived view and physical activity
[117]	2020	Andalusia, Spain	479 respondents between 18 and 64 years	View of urban green spaces from home referred to the possibility of viewing green spaces from any of the home windows	Chi-square tests and a multiple linear regression models used to identify the variables explaining the risk of anxiety and Depression.	Adults who enjoy a view of green spaces from home have a lower risk of anxiety and depression.	Visual stimulation
[5]	2013	the United Kingdom	10168 individuals from the British Household Panel Survey	Local-area green space were derived from the Generalised Land Use Database	Fixed-effects regression approach that estimated the effects of green space based on scores for the same individuals at different points in time and thus controlled for personality and other stable factors.	On average, individuals have both lower mental distress and higher well-being when living in urban areas with more green space.	Neighborhood satisfaction

**Table A2:** Summary of some of the literature research methods employed in retrieved articles.

	Specific Items	Document No.	Mental State Measurement Tool	Experimental Method
Mental State	Poor psychological condition	[33]	Health survey brief form (SF)-36	Cross-sectional
	Mental health	[86]	General health questionnaire (GHQ)-12 and SF-36	Cross-sectional
	Psychological State	[111]	GHQ-30	Cross-sectional
	Mental health	[112]	Mental health scale (MHI)-5	Cross-sectional
	Neighborhood happiness	[114]	General statement and Pearson correlation coefficient	Sampling survey, Linear regression
	Neighborhood satisfaction	[52]	SF-12	Joint significance test
Mood	Anxiety	[118]	Ministry of health database	Cross-sectional
	Anger, confusion, fatigue, and vitality	[44,106,119]	Profile of mood states (POMS) questionnaire	Quasi-experimental Properties (control)
	Depression	[119]	Modified depression scale (MDS)	Cross-sectional
	Fear, happiness, and sadness	[101]	POMS questionnaire	Quasi-experimental Properties (control)
	Positive/negative emotions	[28,37]	Depression and anxiety scale (DASS-21)	Quasi-experimental Properties (control)
	Emotional recovery	[92]	POMS questionnaire	Quasi-experimental
	Self-esteem and general emotional interference	[44]	The diagnostic and statistical manual of mental disorders (DSM-IV)	Cross-sectional
Restorative	Humans’ restoration	[15,82]	Restorative outcome scale (ROS)	Quasi-experimental Properties (control)
	Environment restores mental alertness	[49]	Perceived restorativeness scale (PRS)	Multiple regression (step-wise) analysis
	Behavioral problem	[25]	By assessing children’s internalization and externalization ability	Longitudinal design
	Behavioral problems	[98]	Strengths and difficulties questionnaire (SDQ)	Portrait (queue)
	Psychological distress	[38]	Kessler psychological distress scale (K10)	Cross-sectional
	Perceived stress	[13]	Probability proportionate to size (PSS)	Cross-sectional
	Chronic stress	[100]	Hair cortisol	Cross-sectional
