# Supplementary material for: How Does Urban Green Space Impact Residents’ Mental Health: A Literature Review of Mediators
Source: Int J Environ Res Public Health. 2021 Nov 9;18(22):11746. doi: 10.3390/ijerph182211746 (PMC8621109; doi:10.3390/ijerph182211746)
Supplement: Supplementary file 1 [file ijerph-18-11746-s001.zip › ijerph-1453366-supplementary.pdf]

Supplement

Table S1. Quality assessment tool

NO.	Item	Description	Scale
1	Study Design	Was the study design clearly identified, and is the rationale for choice of design evident?	1: The design was appropriate for the study question<br>0.5: Insufficiently described<br>0: The design was not appropriate for the study question
2	Population - Selection bias	Are the individuals selected to participate in the study likely to be representative of the target population?	1: Likely to be representative<br>0.5: Insufficiently described<br>0: Unlikely to be representative
3	Population –Inclusion bias	Is there evidence of bias in the percentage of selected individuals who provided data for inclusion in the analysis?	1: No evidence of bias<br>0.5: Insufficiently described<br>0: Evidence of bias
4	Green space measure - derivation	Was derivation of the green space variable well described?	1: Derivation of green space measure well described<br>0.5: Insufficiently described<br>0: Derivation of green space measure not described
5	Green space measure - type	Did the green space measure include information on type of green space?	1: Green space measure included information on type of green space<br>0.5: Insufficiently described<br>0: Green space measure did not include information on type of green space
6	Quality of green space	Quality of green space was measured and included in analysis	1: Measured quality of green space<br>0.5: Insufficiently described<br>0: Did not measure quality of green space
7	Mental health measure - derivation	Was derivation of the mental health variable well described?	1: Derivation of mental health measure well described<br>0.5: Insufficiently described<br>0: Derivation of mental health measure not described

8	Statistical methodology	Was an appropriate statistical methodology used?	1: Evidence of appropriate methodology<br>0.5: Insufficiently described<br>0: No evidence of appropriate methodology
9	Outcome - reliable	Were the outcome measures reliable	1: gave the same information over different situations<br>0.5: Insufficiently described<br>0: gave the different information over different situations
10	Outcome - valid	Were the outcome measures valid?	1: the measure assessed what it was intended to measure.<br>0.5: Insufficiently described<br>0: the measure did not access what it was intended to measure.
11	Effect size	Was an effect size (estimate and confidence interval/standard error) reported for green space variable?	1: Effect size reported for green space<br>0.5: Insufficiently described<br>0: Effect size not reported for green space
12	Mediator	Have any mediators been reported?	1: Mediators were reported<br>0.5: Insufficiently described<br>0: No mediators were reported
13	Heterogeneity	Is there any discussion about individual or social characteristics?	1: Heterogeneity was discussed<br>0.5: Insufficiently described<br>0: No Heterogeneity was discussed
14	Level of analysis	Was analysis of green space access in relation to outcome carried out at individual level or at ecological (area) level	1: Individual level<br>0.5: Insufficiently described<br>0: Ecological level

Table S2. Quality assessment score

**Reference No.**	[10]	[52]	[24]	[31]	[35]	[26]	[28]	[114]	[36]	[33]	[37]	[38]	[50]	[39]
Study Design	1	1	1	1	1	1	1	1	1	1	1	1	1	1
Population - Selection bias	0.5	1	0.5	0.5	0.5	0.5	0.5	0.5	1	1	0.5	1	1	0.5
Population –Inclusion bias	0	0	0	0	0.5	0	0.5	0.5	0.5	0.5	0.5	0.5	0.5	0.5
Green space measure - derivation	0.5	0.5	1	1	0.5	1	0	0.5	1	1	1	1	0.5	0.5
Green space measure - type	1	0.5	1	0.5	1	0.5	0.5	0	1	1	1	1	0.5	0.5
Quality of green space	1	0	0	1	0	0	0	0	0	0	0	0	1	0
Mental health measure - derivation	1	1	1	1	0.5	0.5	0.5	1	0.5	0.5	0.5	1	1	1
Statistical methodology	0.5	1	1	1	1	1	1	1	1	1	1	1	1	0.5
Outcome - reliable	0.5	1	1	0.5	0.5	1	0.5	0.5	1	1	1	1	1	1
Outcome - valid	1	0.5	1	1	1	1	0.5	0	1	0.5	0.5	1	0.5	1
Effect size	1	0.5	0.5	1	1	1	1	1	1	1	1	1	1	1
Mediator	0	1	0.5	1	1	0.5	1	1	1	1	1	1	1	1
Heterogeneity	0	0	0	0.5	0.5	1	0	0	1	1	1	1	1	1
Level of analysis	0.5	1	1	1	1	1	1	1	1	1	1	1	1	1
**SUM**	8.5	9	9.5	11	10	10	8	8	12	11.5	11	12.5	12	10.5

**Reference No.**	[25]	[40]	[5]	[98]	[67]	[15]	[46]	[82]	[48]	[49]	[26]	[108]	[44]	[41]
Study Design	1	1	1	1	1	1	1	1	1	1	1	1	1	1
Population - Selection bias	1	1	1	1	1	0.5	0.5	0.5	0.5	1	1	1	1	0.5
Population –Inclusion bias	1	0.5	1	1	0.5	0.5	0.5	0.5	0.5	0.5	0.5	0.5	0.5	0.5
Green space measure - derivation	1	1	0.5	1	0.5	0.5	0.5	0.5	1	0.5	1	1	1	0.5
Green space measure - type	0.5	0	1	0.5	0.5	1	1	1	1	1	0.5	1	0.5	0.5
Quality of green space	0	0	0	0	0	0	0	0	0	0	0	0	1	0
Mental health measure - derivation	1	1	1	1	1	1	1	1	1	1	1	1	1	0.5

Statistical methodology	1	1	1	1	0.5	0.5	1	1	1	0.5	1	1	0.5	1
Outcome - reliable	1	1	0.5	1	1	1	1	1	1	1	1	1	1	1
Outcome - valid	0.5	0.5	1	0.5	0.5	0.5	1	0.5	0.5	1	0.5	0.5	0.5	0.5
Effect size	1	1	1	1	0.5	1	1	1	1	1	1	1	1	1
Mediator	1	1	0.5	0.5	1	0.5	1	0.5	0.5	0.5	1	0	1	0.5
Heterogeneity	1	1	0	1	1	0	0	0.5	0	0.5	1	1	1	0.5
Level of analysis	1	1	1	1	1	1	1	1	1	1	1	1	1	1
**SUM**	12	11	10.5	11.5	10	9	10.5	10	10	10.5	11.5	11	12	9

**Reference No.**	[115]	[116]	[42]	[51]	[45]	[43]	[29]	[117]	[47]	[22]	[17]	[27]	[76]	[32]
Study Design	1	1	1	1	1	1	1	1	1	1	1	1	1	1
Population - Selection bias	1	1	1	1	1	1	1	0.5	0.5	1	1	1	1	1
Population –Inclusion bias	0.5	0	0.5	0.5	0.5	0.5	0.5	0.5	0.5	0.5	0.5	0.5	0.5	0
Green space measure - derivation	1	0.5	1	1	0.5	0.5	1	1	1	1	0.5	1	1	1
Green space measure - type	1	0.5	1	0	1	1	1	1	1	1	0.5	0.5	0.5	0.5
Quality of green space	0	0	0.5	0	0	1	0	0	0	0	0	0	0	0
Mental health measure - derivation	0.5	1	1	1	1	0.5	1	1	1	1	1	1	1	1
Statistical methodology	1	1	1	1	1	1	1	1	1	1	1	1	1	1
Outcome - reliable	1	1	1	1	1	1	1	1	1	1	1	1	1	0.5
Outcome - valid	1	1	0.5	0.5	0.5	0.5	0.5	0.5	1	0.5	0.5	1	0.5	1
Effect size	1	1	1	1	1	1	1	1	1	1	1	1	1	1
Mediator	0	0.5	1	1	1	1	1	1	0	0	0.5	0.5	1	1
Heterogeneity	1	0.5	1	0	1	0	1	0	0	0.5	0	0	0	0
Level of analysis	1	1	1	1	1	1	1	1	1	1	1	1	1	1
**SUM**	11	10	12.5	10	11.5	11	12	10.5	10	10.5	9.5	10.5	10.5	10
